# Comparative Analysis of Chronic Diseases and Depression Symptoms Between Participants and Non-Participants of Physical Activity Among Chinese Older Adults in Urban and Rural Areas

**DOI:** 10.3390/healthcare13131545

**Published:** 2025-06-28

**Authors:** Ziwei Liang, Chaoqi Li, Sihong Sui, Zhimin He, Yi Ren, Zixiang Zhou, Kyungsik Kim

**Affiliations:** 1 Department of Sport & Leisure Studies, Hoseo University, Asan-si 31499, Republic of Korea; ww454092273@gmail.com (Z.L.); jungle9031@gmail.com (C.L.); suisihong@gmail.com (S.S.); 20235617@vision.hoseo.edu (Y.R.); 2 School of Physical Education, Gansu University of Political Science and Law, Anning West Road, Lanzhou 730070, China; hzm6957@gsupl.edu.cn; 3 School of Physical Education, Hunan University of Science and Technology, Taoyuan Road, Xiangtan 411201, China

**Keywords:** physical activity, chronic diseases, depression symptoms, older adult, disparities, urban, rural

## Abstract

**Introduction**: Based on data from the China Health and Retirement Longitudinal Study 2020 (CHARLS 2020), we analyzed the effects of physical activity (PA) on chronic diseases and depression symptoms in older adults in urban and rural areas and examined differences by residential location. **Methods**: A total of 5481 individuals aged 65 years and above were selected from the CHARLS 2020 dataset. Descriptive statistics, chi-square tests, two-way analysis of variance, and Pearson’s correlation analysis were used to examine the influence of different intensities of PA on chronic diseases and depression symptoms. According to PA recommendations, PA participants were individuals who engaged in PA two or more times per week, while non-participants engaged in PA fewer than two times per week. **Results**: Urban and rural older adults showed different patterns in PA participation and its health impacts. Urban residents were more likely to engage in high-intensity PA, which was related to lower prevalence of chronic diseases and fewer depressive symptoms; moderate-intensity PA was also effective in relieving depressive symptoms. In contrast, rural residents primarily participated in low-intensity PA, which had some effect in alleviating depression symptoms but limited impact on chronic diseases. **Conclusions**: Public health interventions should be tailored to regional differences. In rural areas, the promotion of appropriate PA programs is essential to improve overall health, while urban areas should emphasize mental health strategies, social engagement, and support network development.

## 1. Introduction

As China’s aging population continues to increase, health issues among older adults have become a critical challenge for the public health sector. According to a report by the National Health Commission of the People’s Republic of China, a substantial proportion of older adults have chronic diseases, with 78% reporting at least one chronic condition [1]. Chronic conditions, including hypertension, diabetes, dyslipidemia, heart disease, and stroke, are particularly prevalent among older adults, with incidence rates rising markedly with age [2,3,4]. Furthermore, depression is common among older adults and closely associated with insufficient social support, loneliness, and gradual deterioration of physical health [5,6]. In China, the Healthy China Action (2019–2030) is a long-term plan to promote national health and disease prevention. The goal is to strengthen the health service system for the elderly, create an environment for promoting older adult health, and reduce the premature mortality rate due to major chronic diseases [7]. While this policy has helped improve older adult health, stark differences exist between urban and rural areas. Urban elderly have greater access to health promotion services than rural elderly [8].

Although Healthy China Action has made progress in preventing chronic diseases and promoting mental health in older adults, health inequalities between urban and rural older adults demand attention. These challenges are exacerbated by the uneven distribution of healthcare resources between urban and rural areas, limited health management capacity at the grassroots level, and fragmentation of disease prevention and control systems, all of which hinder improvement in the health of older adults [9,10].

Many older adults fail to engage in adequate physical activity (PA), which is a significant contributing factor to various health issues [11,12]. Studies have indicated that regular PA can substantially lower hypertension incidence, enhance blood glucose control in diabetes, and mitigate depressive symptoms [13]. PA, through mechanisms such as the enhancement of cardiovascular function and regulation of the neuroendocrine system, has been demonstrated to be an effective intervention for preventing chronic diseases and improving the mental health of older adults [14,15]. In addition to its role in chronic disease prevention, PA also plays a crucial role in preserving and enhancing mental well-being, thereby offering dual benefits to older adults. Incorporating regular PA into the daily routine of older adults is a critical strategy for mitigating chronic diseases and mental health disorders. Mental health conditions, such as depression and anxiety, can significantly worsen the physical health of older adults [16,17]. These conditions may impair immune function and contribute to the progression of chronic diseases [18]. PA stimulates the release of endorphins, which enhances mood and alleviates stress [19]. Furthermore, regular exercise fosters social interaction and reduces feelings of loneliness, thereby promoting overall mental well-being [20].

However, disparities in health issues between older adults living in urban and rural areas should not be overlooked. Compared to rural adults residing in urban areas, older adults generally benefit from greater access to well-developed sports facilities, more comprehensive health information, and better economic resources, all of which contribute to increased participation in PA and advancement in health management practices [21]. In contrast, older adults in rural areas often experience challenges, such as limited transportation infrastructure, low health awareness, and inadequate medical resources, resulting in reduced engagement in PA and less effective disease management [22]. Although older adults in urban areas benefit from improved access to healthcare services and medical care, their rural counterparts face significant challenges in accessing similar resources. Therefore, a comprehensive analysis of urban–rural differences is essential in studying the health issues of older adults to gain a thorough understanding of these disparities and establish a foundation for more targeted intervention strategies.

Although existing research has examined the relationship between PA, chronic diseases, and depression symptoms, several limitations persist in current studies. Many studies do not sufficiently account for urban–rural disparities, resulting in an incomplete understanding of health outcomes among older adults. These disparities are not simply due to differences in PA participation rates, but rather to factors such as lifestyle, access to health resources, economic conditions, and social support [23]. In other words, there may be inequalities in older adults’ opportunities for physical activity between urban and rural areas, and differences in the experience of the effects of participation in PA, such as chronic diseases and depression [24,25]. Failure to account for these urban-rural disparities in research often results in findings that are predominantly applicable to urban populations, thereby hindering the development of effective intervention strategies for rural older adults and diminishing the overall efficacy and relevance of public health policies.

Moreover, most existing studies have primarily examined individual diseases and failed to adequately account for multiple chronic diseases. In older adults, common chronic diseases, including hypertension, diabetes, and heart disease, frequently co-occur, and their interactions may exacerbate mental health disorders such as depression and anxiety [26,27,28,29]. Consequently, studies focusing solely on individual diseases may fail to highlight the comprehensive effects of PA in addressing multiple health problems. PA, through its multifaceted benefits, not only enhances the management of various chronic diseases but also plays a crucial role in improving the emotional and mental health of older adults [30,31].

Existing evidence has further highlighted the correlations between residential areas, number of PA days, chronic diseases, and depression symptoms among older adults. Researchers have reported that urban or rural residency can significantly influence the relationship between PA frequency and health outcomes. Specifically, urban older adults generally exhibit higher chronic disease prevalence but lower levels of depression than those observed among rural older adults [25]. Additionally, the number of days engaged in regular PA is closely related to a reduced chronic disease incidence and improved mental health status [32,33]. Researchers have emphasized that the association varies by residential area; in urban populations, the number of PA activity days is more strongly and negatively correlated with depression, whereas in rural populations, a stronger negative correlation with the prevalence of chronic diseases is observed [34,35,36]. These differential patterns suggest that the strength and direction of the correlations between PA frequency and health outcomes are context-dependent and shaped by the distinct health profiles and environmental conditions of urban and rural older adults.

Finally, numerous studies have used small-scale sample data, thereby restricting the generalizability of their findings. For instance, variations in health status and PA between urban and rural older adult populations are shaped by multiple factors, including regional differences, cultural backgrounds, and economic conditions [37,38]. If research relies solely on specific regions or limited sample sizes, the findings may not accurately represent the health status of older adult populations at the national level, particularly in countries with pronounced urban–rural disparities. Small sample sizes can result in inaccurate assessments of the impact of PA on the health of older adults, thereby diminishing the effectiveness of intervention strategies.

To overcome the limitations of existing research, this study utilized data from the China Health and Retirement Longitudinal Study (CHARLS), which encompasses a broad sample of older adults and offers a nationally representative sample. Through an urban–rural stratified analysis of older adult populations, this study aimed to more precisely assess the impact of urban–rural disparities on PA participation and health outcomes, thereby addressing the issue of insufficient consideration of these differences in prior research. Furthermore, this study extends beyond the examination of a single disease to consider the simultaneous presence of multiple chronic conditions, including hypertension, diabetes, heart disease, stroke, and depression symptoms. This study further evaluated the multifaceted benefits of PA in mitigating these health concerns, thereby addressing the research gap identified in prior studies that predominantly focused on individual diseases. In addition, this study sought to bridge several gaps in existing research by employing large-scale, nationally representative data to compare the prevalence of chronic diseases and depression among urban and rural older adults who participate in and do not participate in PA. The findings of this study can elucidate the distinct effects of PA on chronic diseases and mental health among urban and rural older adult populations, thereby providing theoretical support for policymakers in developing targeted intervention strategies.

## 2. Materials and Methods

### 2.1. Research Design

We submitted a research proposal to the National Development Institute of Peking University and obtained approval to use data from the CHARLS 2020 for our study. This data is a nationally representative longitudinal survey project organized by the National Institute of Development Studies of Peking University, which mainly covers information about the Chinese adult population aged 45 years and older [39]. Given that this study focuses on the health status of the elderly population aged 65 years and older, we screened and extracted data from the CHARLS 2020 database from a sample of age-eligible individuals for subsequent analysis and discussion.

Using a cross-sectional design, this study used secondary data from the CHARLS 2020 survey to compare and analyze PA participation and the experience rate of chronic diseases and depression symptoms among older adults aged 65 years and older by residential area (urban vs. rural).

**Figure 1:**
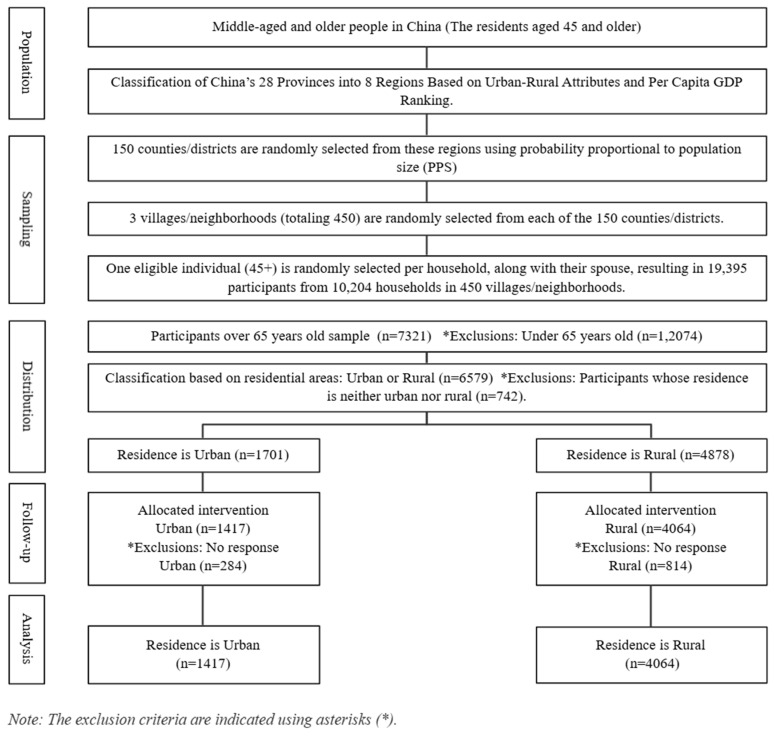
Flow diagram of the study participants.

The CHARLS 2020 survey included 19,395 participants. Of these, 7321 were aged 65 years or older. This study examined differences in the experience rate of chronic diseases and depression symptoms among older adults based on their participation in PA, with a focus on urban and rural populations. Of the 7321 older adults, 4878 lived in urban areas, and 1701 lived in rural areas. However, 742 participants who lived in ‘unspecified geographic areas’ (e.g., urban–rural transition areas and special areas) were excluded from the analysis due to their limited representation and the unique social contexts they represented: peri-urban areas, government-led resettlement areas, policy-based migrant villages, central aged care facilities, remote military reclamation areas, and isolated mining or forestry areas. Subsequently, 1098 participants (284 urban, 814 rural) who did not respond to the main PA or health variables were also excluded. The final sample consisted of 5481 participants, 1417 from urban areas and 4064 from rural areas. Detailed information is presented in Figure 1, with the exclusion criteria indicated using asterisks.

To ensure the representativeness of the sample, the CHARLS 2020 survey covered 28 provinces, 150 regions, and 450 towns and urban communities across China, providing a comprehensive representation of the older adult population. The survey employed a multistage probability proportional to size random sampling method (county/district → village/community → household) based on implicit stratification, with stratification indicators including region, urban–rural classification, and gross domestic product per capita [40]. Data was collected through face-to-face interviews using a computer-assisted personal interviewing system.

The CHARLS 2020 study was approved by the Biomedical Ethics Committee of Peking University (Approval Number: IRB00001052-11015), and written informed consent was obtained from all participants or their designated proxy respondents. To further strengthen compliance with research ethics, this study also received additional ethical approval from the Institutional Review Board of Hunan University of Science and Technology, the affiliated institution of a co-researcher (Approval Number: HT2025002).

### 2.2. Measures

Demographic characteristics included sex, age, type of residence, educational level, marital status, poverty status, and smoking and drinking habits. Age was categorized into the following groups: 65–69 years, 70–74 years, 75–79 years, and 80 years and older. Educational level was classified as less than primary school, middle school, high school, or higher than college. Residence types were divided into urban and rural. Marital status was categorized as married (with a spouse present), separated, divorced, widowed, or never married. Smoking habits were classified as currently smoking, formerly smoked, or never smoked. Drinking habits were categorized as drinking more than once a month, drinking less than once a month, or not drinking.

We used a local shortened version of the globally recognized International Physical Activity Questionnaire, which is a widely used tool for assessing an individual’s PA level. PA was categorized into three types: vigorous intensity, moderate intensity, and mild intensity [41]. Vigorous-intensity activities are activities that cause shortness of breath. Examples include carrying heavy loads, digging, hoeing, aerobic workouts, cycling at high speeds, and riding cargo bicycles or motorcycles. The number of vigorous-intensity PA days refers to the days in the past week when the respondent experienced shortness of breath during exercise lasting more than 10 min (e.g., “During a usual week, on how many days did you engage in vigorous activities for at least 10 min?”). The duration of vigorous PA was recorded in hours and minutes per day. Moderate-intensity activities are activities that cause a faster breathing pace than usual. Examples include carrying light loads, cycling at a normal pace, mopping, practicing Tai Chi, and brisk walking. Respondents answered questions such as, “During a usual week, on how many days did you engage in moderate activities for at least 10 minutes?” and “How much time do you usually spend doing moderate activities on one of those days?” Low-intensity activities included walking from one location to another at work or home and walking for leisure, exercise, sports, or entertainment. In this study, PA participation was categorized into high-, moderate-, and low-intensity activities. PA participants were defined as individuals who engaged in PA two or more times per week, whereas non-participants were defined as those who engaged in PA less than two times per week. These criteria are based on statistics from the Ministry of Culture, Sports, and Tourism of Korea. This department conducts surveys about sport participation every year, using a participation rate of at least twice a week as the main criterion. This recommendation suggests that engaging in PA more than twice a week is necessary to achieve the benefits of exercise.

In this study, depression was assessed based on participants’ experiences of depressive symptoms. The 10-item Center for Epidemiologic Studies Depression (CESD-10) scale was used to assess depression symptoms. This scale has been validated as a reliable and effective tool for assessing mental health in older Chinese populations [42,43]. The CESD-10 consists of 10 items that reflect common depressive symptoms, such as feeling down, loss of interest, sleep disturbances, loneliness, and lack of energy. Respondents rated the frequency of each symptom over the past week on a four-point scale: 0 (rarely or none of the time [<1 day]), 1 (some or few times [1–2 days]), 2 (occasionally or a moderate amount of the time [3–4 days]), and 3 (most or all of the time [5–7 days]). For the two positive affect items (“I was happy” and “I felt hopeful about the future”), the scoring was reversed. The total CESD-10 score ranged from 0 to 30, with higher scores indicating more severe depressive symptoms. A score of 10 or higher is generally indicative of depression risk [44,45].

The chronic disease rate refers to the prevalence of long-term medical conditions among participants, including hypertension, diabetes, heart disease, and stroke. These conditions were assessed through self-reported diagnoses by healthcare providers. Specifically, the participants were asked, “Have you ever been diagnosed with hypertension, diabetes, heart disease, or stroke by a doctor?” This ensured that only medically confirmed diagnoses were included in the analyses.

### 2.3. Statistical Analysis

Although we did not calculate a priori sample size due to the use of secondary data, we performed post hoc power analysis using G*Power 3.1. The result revealed that the sample size was sufficient to detect meaningful effects with high statistical confidence.

Statistical analyses were conducted using IBM SPSS Statistics for Windows, Version 26.0 (IBM Corp., Armonk, NY, USA), to examine differences in chronic diseases and depression symptoms between PA participants and non-participants in urban and rural residential areas.

To assess the differences in PA participation of varying intensities and the prevalence of chronic diseases by residential area, we performed chi-square tests. The chi-square test was used to determine the relationship between categorical variables (e.g., residential area) and the presence or absence of chronic diseases. All statistical tests were performed with a significance threshold set at *p* < 0.05 to ensure the robustness of the findings.

We performed two-way analysis of variance tests to assess the main effects of PA level and residential area on depression scores. In addition, we investigated the interaction between PA level and residential areas to determine whether the relationship between PA and depression symptoms differed according to residential environment. We compared depression scores between PA participants and non-participants at each PA level (high-, moderate-, and low-intensity) in both urban and rural populations. When significant differences were found, post hoc tests were performed to further explore these changes.

In addition, correlation analyses were performed to investigate the relationships between urban residence, PA level, chronic diseases, and depression symptoms. Pearson’s correlation coefficients were calculated to quantify the strength and direction of the associations between these variables. The significance level for the correlation analysis was set at *p* < 0.05; for highly significant correlations, a more stringent threshold of *p* < 0.001 was applied. This analysis was targeted at gaining insight into the association between urban residence and chronic disease prevalence, and the effects of PA on chronic diseases and depression symptoms.

We used Cramer’s v to verify the effect size (ES) of the cross-analysis. The closer this value is to 0, the weaker the correlation, and the closer it is to 1, the stronger the correlation. We used Partial Eta Squared to verify the effect size of the two-way ANOVA; this value should be over 0.06 to indicate that the variable or interaction effect explains about 6% of the variation in the dependent variable.

The statistical significance for all the statistical tests was set at *p* < 0.05, which is a commonly accepted threshold for statistical analysis.

## 3. Results

### 3.1. Differences in Chronic Diseases by Residential Areas

#### 3.1.1. Differences in Hypertension by Residential Areas

According to the findings (see Table 1), vigorous-intensity PA was associated with a noticeably lower hypertension experience rate among urban participants than among non-participants; however, this difference was not statistically significant (4.5%) (*p* > 0.05). In contrast, in rural areas, participants had a significantly lower hypertension experience rate (15.0%) than non-participants (36.8%) (*p* < 0.001, ES = 0.095). Regarding moderate-intensity PA, urban participants had a lower hypertension experience rate than non-participants, but the difference was not statistically significant (*p* > 0.05). However, in rural areas, participants (21.3%) showed a significantly lower hypertension experience rate than non-participants (30.5%) (*p* < 0.001, ES = 0.070). For low-intensity PA, participants in both urban and rural areas exhibited a higher hypertension experience rate than non-participants; however, these differences were not statistically significant (*p* > 0.05). Overall, these findings suggest an inverse relationship between PA intensity and hypertension experience rate, highlighting that vigorous- and moderate-intensity PA may be particularly effective in reducing the risk of hypertension.

#### 3.1.2. Differences in Diabetes by Residential Areas

According to the findings (see Table 1), vigorous-intensity PA was associated with a lower diabetes experience rate among urban participants than among non-participants; however, this difference was not statistically significant (*p* > 0.05). In contrast, in rural areas, the diabetes experience rate was significantly lower among participants (4.7%) than among non-participants (11.7%) (*p* < 0.01, ES = 0.046). For moderate-intensity PA, participants in both urban and rural areas exhibited a lower diabetes experience rate than non-participants; however, these differences were not statistically significant (*p* > 0.05). For low-intensity PA, participants in both urban and rural areas demonstrated a higher diabetes experience rate than non-participants; however, this difference was not statistically significant (*p* > 0.05). These findings suggest an inverse relationship between vigorous-intensity PA and diabetes experience rate in rural areas, indicating that vigorous-intensity PA might play the most effective role in reducing the risk of diabetes.

#### 3.1.3. Differences in Heart Disease by Residential Areas

According to the findings (see Table 1), vigorous-intensity PA was associated with a statistically significant reduction in the heart disease experience rate in urban participants compared to non-participants (35.9%) (*p* < 0.001, ES = 0.086). Similarly, in rural areas, the heart disease experience rate among participants was significantly lower than among non-participants (7.3%) (*p* < 0.001, ES = 0.089). For moderate-intensity PA, the heart disease experience rate among urban participants was significantly lower than among non-participants (22.4%) (*p* < 0.01). In contrast, in rural areas, participants with moderate-intensity PA exhibited a lower heart disease experience rate (11.7%) than non-participants (15.8%); however, this difference was not statistically significant (*p* > 0.05). Regarding low-intensity PA, participants in both urban and rural areas demonstrated higher rates of heart disease than non-participants, but these differences were not statistically significant (*p* > 0.05). These findings highlight the significant association between PA intensity and heart disease experience rate, suggesting that vigorous-intensity PA and, to a lesser extent, moderate-intensity PA, might play a particularly effective role in preventing heart disease.

#### 3.1.4. Differences in Stroke by Residential Areas

According to the findings (see Table 1), vigorous-intensity PA was associated with a lower stroke experience rate among participants than among non-participants in urban areas; however, this difference was not statistically significant. In contrast, in rural areas, participants with vigorous-intensity PA (3.0%) had a significantly lower stroke experience rate than non-participants (8.7%) (*p* < 0.001, ES = 0.061). For moderate-intensity PA, participants in urban areas (4.6%) and rural areas (4.3%) exhibited a lower stroke experience rate than non-participants (8.2% and 7.4%, respectively). These differences were statistically significant in both urban and rural areas (*p* < 0.001, ES = 0.079, 0.056). Regarding low-intensity PA, participants in both urban and rural areas demonstrated a higher stroke experience rate than non-participants; however, these differences were not statistically significant (*p* > 0.05). These findings suggest that vigorous- and moderate-intensity PA might play significant roles in reducing the risk of stroke.

### 3.2. Differences in Depression Symptoms by Residential Areas

According to these findings (see Table 2), vigorous-intensity PA was associated with lower depression levels among participants than non-participants in urban areas. However, in rural areas, participants exhibited slightly higher depression levels than non-participants. Nevertheless, the main effects of vigorous PA, residential area, and their interactions were not statistically significant (*p* > 0.05). For moderate-intensity PA, depression symptom levels among participants (M = 7.170) were lower than among non-participants (M = 8.290) in urban areas, whereas in rural areas, participants (M = 10.370) had slightly higher depression symptom levels than non-participants (M = 10.270). The main effects of moderate PA and residential areas on depression symptom levels were not statistically significant (*p* > 0.05); however, the interaction effect between moderate PA and residential areas was statistically significant (*p* < 0.01, ES = 0.002). Regarding low-intensity PA, depression symptom levels among participants (M = 7.410) were significantly lower than among non-participants (M = 9.790) in urban areas. In rural areas, participants (M = 10.140) had slightly lower depression symptom levels than non-participants (M = 10.730). Although the main effects of low PA and residential area were not statistically significant (*p* > 0.05), their interaction effect was statistically significant (*p* < 0.01, ES = 0.002). These findings suggest that the relationship between PA intensity and depression symptoms might vary by residential area. In particular, the interaction between residential area and PA appeared to influence depression symptom levels in the moderate and low PA groups.

### 3.3. Correlation Between Urban Residence, PA, Chronic Diseases, and Depression Symptoms

When examining the correlation between urban residence, PA, chronic diseases, and depression symptoms in older adults (see Table 3), we found that urban area residents exhibited a higher diabetes experience rate than rural area residents (*r* = 0.103, *p* < 0.001). Conversely, urban residency (vs. rural residency) was related to lower occurrence of depression symptoms (*r* = −0.169, *p* < 0.001).

As the days of vigorous-intensity PA increased, the experience rate for hypertension (*r* = −0.095, *p* < 0.001), diabetes (*r* = −0.071, *p* < 0.001), heart disease (*r* = −0.112, *p* < 0.001), and stroke (*r* = −0.059, *p* < 0.001) decreased. However, a weak but statistically significant positive correlation emerged between days of vigorous-intensity PA and depression symptoms (*r* = 0.030, *p* < 0.05), suggesting a complex relationship that warrants further investigation. Similarly, an increase in days of moderate-intensity PA was associated with a reduction in the experience rate for hypertension (*r* = −0.069, *p* < 0.001), heart disease (*r* = −0.033, *p* < 0.05), stroke (*r* = −0.062, *p* < 0.001), and depression symptoms (*r* = −0.027, *p* < 0.05). In contrast, a significant negative correlation emerged only between days of low-intensity PA and depression symptoms; as days of low-intensity PA increased, depression symptoms decreased (*r* = −0.089, *p* < 0.001).

These results suggest that high-intensity PA relates to reduced rates of hypertension, diabetes, heart disease, and stroke. In addition, when examining the relationship between PA level and depression symptoms, low-intensity PA had a stronger impact than moderate-intensity PA. Although these correlations were statistically significant, they were generally low.

## 4. Discussion

This study utilized data from the CHARLS 2020 survey to examine the associations between PA of varying intensities (vigorous, moderate, and low) and a range of health outcomes, including chronic conditions (hypertension, diabetes, heart disease, and stroke) and depression symptoms, among urban and rural older adults in China. It compared the health statuses of physically active and inactive older adults across these settings to identify disparities in PA level and health indicators. Additionally, correlation analyses were conducted to explore the different health benefits of PA between urban and rural populations, thereby elucidating the role of regular PA in promoting the physical and mental health of older adults in China. These findings provide new evidence-based support for the development of regionalized, differentiated health intervention strategies for older adults.

We found a statistically significant correlation between PA level and chronic disease experience rate, a finding that is consistent with existing literature findings. Studies have shown that regular participation in PA can help reduce the probability of developing various chronic diseases, including hypertension, diabetes, heart disease, and stroke, in older adults [46]. Notably, previous findings from multiple studies show a negative correlation between moderate to vigorous PA (MVPA) and cardiac metabolic health [47]. For example, You et al. [48] found that older Chinese adults who regularly participated in moderate-to-vigorous-intensity PA had a significantly lower risk of hypertension than those who only performed low-intensity exercise. Similarly, a national cross-sectional study conducted by Huang and Lu [49] revealed that high-intensity PA negatively correlated with the incidence of chronic diseases such as hypertension, diabetes, heart disease, and stroke. Furthermore, a meta-analysis by Lee, Folsom, and Blair [50] pointed out that approximately 20% of people engage in moderate-intensity PA and 27% in high-intensity PA, both of which are associated with a reduced risk of chronic diseases. These findings are consistent with the broader epidemiological literature on the protective effects of PA against chronic diseases.

Moreover, this study differentiated between the effects of varying PA intensities, a distinction that has been noted in previous research. Moderate-intensity PA is significantly related to improved health, while low-intensity PA has fewer health benefits [51]. Consistent with this, Li [52] reported a positive association between low-intensity PA and the prevalence of type 2 diabetes among urban older adults in China, which was interpreted as a possible consequence of increased low-intensity PA following a diabetes diagnosis in accordance with medical recommendations [53]. Similarly, Booths et al. [54] observed that low-intensity PA alone may be insufficient to prevent chronic diseases and, in cross-sectional analyses, was more frequently observed among individuals already diagnosed with chronic conditions. In contrast, MVPA shows a clearer correlation with health protection [55].

However, the health benefits of different PAs of varying intensities differed between older adults living in urban and rural areas. In rural areas, the correlation between MVPA and blood pressure control was more significant, while in urban areas, this type of activity more strongly correlated with the chronic disease experience rate. This difference might relate to background factors such as lower health literacy levels, lack of medical resources, and poor medication adherence among rural populations [56]. Consequently, when rural older adults begin participating in regular MVPA, they may exhibit more substantial improvements owing to a lower baseline health and wider margin for enhancement [57]. In contrast, older adults living in urban areas usually have better access to medical services and equipment, and health education for older adults is often conducted in urban areas [58]. These factors help them to manage their health more effectively and prevent chronic diseases [59]. Our findings suggest that public health strategies should fit the different living environments in urban and rural areas. For example, urban communities can promote moderate-intensity PA such as Tai Chi, Qigong, and square dancing, while rural areas should combine local cooperatives and village service centers to promote collective sports with cultural characteristics in order to enhance the sustainability and actual effectiveness of participation.

We also found a correlation between PA and depression symptoms, with differences between urban and rural areas. Among urban older adults, those who actively participated in PA had significantly lower depression symptoms than those who did not participate. However, this correlation was weaker among rural older adults. This may be attributed to factors commonly found in rural areas, such as social isolation, inadequate access to mental health services, and greater economic stress [60]. A growing body of empirical evidence suggests that regular engagement in PA can enhance psychological well-being and reduce the risk of depression in later life [61,62]. However, the magnitude of its effects might be due to factors such as the environment and individual psychological resources. Specifically, moderate-intensity PA was associated with a significant reduction in depressive symptoms among older urban adults [63], whereas no statistically significant effect was found among their rural counterparts [64]. Likewise, low-intensity PA was linked to modest mental health benefits in urban areas but had a negligible impact in rural regions [65].

In urban environments, older adults frequently participate in activities such as brisk walking, organized Tai Chi sessions, Qigong, and public square dancing, which not only promote cardiovascular health but also facilitate social engagement, which jointly contributes to a reduced risk of depression [66]. In contrast, PAs among rural older adults typically consist of labour-intensive activities, including planting crops, chopping wood, and walking on a hilly terrain. Although these activities entail physical effort, they may not address the underlying psychological stress, feelings of social isolation, or feelings of loneliness [67]. Consequently, even when rural older adults attain moderate levels of PA, the absence of a supportive psychosocial context may attenuate the mental health benefits typically associated with PA [68]. This explanation aligns with prior findings by Jin, Liu, and Niyomsilp [61], who reported that the buffering effect of PA on depressive symptoms was primarily observed among older urban adults, with limited benefits observed in rural populations (PA had a significant effect on alleviating depressive symptoms in urban older adults, whereas in rural areas this benefit appeared to be more limited). Similarly, Shvedko et al. [69] highlighted that when developing PA interventions, managers should take environmental conditions into account, considering key factors such as social support and resource accessibility.

Correlation analysis revealed that the frequency of older adults participating in MVPA negatively correlated with chronic disease experience rate (e.g., hypertension and diabetes) and the presence of depression symptoms [70,71,72]. Even a weak correlation between PA level and either chronic disease or depression symptom experience rate has important policy implications. In this case, encouraging PA among older adults could lead to improved health. One plausible explanation for this disparity is that PA among rural older adults in China primarily stems from routine labor-intensive tasks such as farming. Although frequent, such activities may not consistently meet the intensity thresholds necessary to confer optimal protective effects [73]. Furthermore, persistent economic and healthcare limitations contribute to higher rates of chronic diseases and depression in rural populations [74]. Consequently, when rural older adults engage in higher-intensity PA, the resultant health benefits may be more pronounced [75]. In contrast, older adults in urban settings, compared with those in rural settings, generally engage in low PA. They mostly participate in PA as recreational or leisure, wherein even moderate-intensity exercises can yield meaningful health improvements [68,76].

In addition, we found a nonlinear relationship between the frequency of PA participation among older adults and the presence of depression symptoms. That is, moderate-frequency, moderate-intensity PA had a stronger effect on reducing depression symptoms, while frequent high-intensity activities related to mild depressive symptoms in certain populations, particularly among urban older adults. This finding might indicate that some older adults use PA as a means of coping with physical problems such as obesity or chronic diseases, inadvertently adding to the mental burden [77,78]. These findings emphasize the complexity PA faces when used as a mental health intervention tool, and that its intervention effects are not universally applicable, but are influenced by a combination of multiple factors, such as an individual’s motivation to participate, the social environment, and physical adaptability [79]. The present study further suggests that there are differential effects of PA frequency and intensity in promoting physical and mental health. Specifically, regular participation in high-intensity PA by rural older adults significantly reduces the incidence of chronic diseases, whereas regular moderate-intensity PA by urban older adults has a more pronounced positive effect on alleviating depression [61]. Therefore, future interventions for older adults should be geographically designed according to urban–rural differences [80]. For instance, for rural areas, older adults may be encouraged to participate in moderate-to-high-intensity PA multiple times per week. In contrast, in urban areas, moderate-frequency and appropriate-intensity PA is recommended to minimize the physical load and risk that may be associated with high-intensity exercise [81].

In summary, the study findings are consistent with the broader body of literature, both within China and internationally, demonstrating that adequate PA yields significant benefits for older adults by preventing major chronic diseases and enhancing mental health. Moreover, this study contributes to existing research by revealing the differential effects of varying activity intensities observed in urban and rural older populations, thereby offering more nuanced evidence to guide the development of future health promotion strategies targeting older adults. Notably, this descriptive study based on cross-sectional data has limited explanatory power. Moreover, missing data might have affected the sample’s representativeness. Because the results rely on self-reported measures of PA and depression, scholars should consider incorporating objective measures and structured interviews to enhance the accuracy and reliability of the data. Therefore, future studies should explicitly address such relationships, particularly considering how variations in residential settings influence the health of older adults, thereby informing targeted and effective intervention strategies. Furthermore, public health interventions should be tailored to the distinct characteristics and needs of urban and rural older adult populations, encouraging daily PA of appropriate intensity to optimize its preventive effects on chronic diseases and promotive effects on mental well-being.

Our findings help clarify the relationship between PA and chronic disease and depression symptoms in urban and rural elderly people through chi-square testing, two-way ANOVA, and correlation analysis, none of which were clearly identified in previous studies. However, these analyses do not control for confounding variables that might affect disease and depression. In future studies, scholars should set up and analyze a multiple regression model to control for variables such as sex, age, education level, and health behaviors.

## 5. Conclusions and Implications

We explored the relationship between PA, chronic diseases, and depression symptoms among urban and rural older adults using the CHARLS 2020 data. The findings point to several key insights. The differentiated effects of PA across regions suggest that a one-size-fits-all public health approach is insufficient. In rural areas, PA contributes more significantly to the prevention and management of chronic diseases (e.g., hypertension and diabetes), whereas in urban areas, PA plays a more prominent role in alleviating depression symptoms and enhancing mental well-being. These disparities underscore the necessity of designing PA interventions that are sensitive to local environmental, infrastructural, and social contexts. 

In rural settings, integrating PA with existing social networks and strengthening community cohesion might amplify its benefits, while urban strategies should focus on reducing environmental stress and promoting accessible, stress-relieving forms of PA for older adults. Nonetheless, our study has limitations. The use of cross-sectional data restricts causal inference, and reliance on self-reported measures might introduce bias. Furthermore, we did not account for contextual factors such as access to PA facilities or neighborhood environments, which might influence outcomes. Overall, the findings highlight the importance of aligning PA promotion strategies with the specific characteristics of urban and rural environments to optimize health outcomes for aging populations.

From a theoretical perspective, the findings highlight context-specific health patterns of association between PA, chronic diseases, and depression symptoms among older adults in urban and rural areas. In previous studies, scholars have often overlooked the dual impact of PA on physical and mental health across different residential environments, particularly the nuanced differences between urban and rural populations. In response, we differentiated the health benefits of PA by intensity level and incorporated residential context as a critical moderating variable, thereby revealing complex interaction effects. Using data from the nationally representative CHARLS 2020, we moved beyond the traditional single-disease perspective and provided a comprehensive analysis of the co-occurrence of multiple chronic conditions and depression symptoms. Importantly, the findings suggest that MVPA was particularly effective in enhancing cardiovascular health among rural older adults, whereas mental health benefits were more evident in urban settings. This divergence provides new theoretical insights into the environment-specific dynamics of health outcomes and highlights the importance of refining intervention models by incorporating spatial and social determinants.

From a practical perspective, the findings have significant implications for public health interventions, older adult care services, and community-based PA programs. Context-specific intervention strategies depend on the different health benefits of PA in urban and rural settings. Specifically, the results highlight the importance of enhancing chronic disease prevention by expanding access to structured MVPA programs in rural areas, where healthcare resources are scarce and the chronic disease burden remains substantial. From a policy perspective, in urban areas, where social isolation and psychological stress are more prevalent, promoting low-to-moderate intensity PA that fosters social engagement might be more effective in alleviating depression symptoms among older adults. Moreover, the observed positive correlation between specific PA intensities and mental health outcomes suggests that future interventions should consider the physical benefits and the psychosocial mechanisms involved in PA participation, including motivation, perceived stress, and social support. In rural areas, existing community organizations (e.g., agricultural cooperatives) can play a vital role in the dissemination and expansion of PA programs. Both national and local governments should prioritize support for marginalized rural older adult populations by enhancing access to PA and sports programs. Consequently, local health authorities and community sports organizations should actively engage in the development and dissemination of PA programs tailored to regional characteristics. For future research, our findings provide a basis for exploring how spatial, social, and psychological factors jointly influence PA effectiveness, calling for longitudinal and intervention-based studies across settings.

## Figures and Tables

**Table 1:** Differences in chronic diseases.

Diseases	Residential Area	Vigorous PA		Moderate PA		Low PA		Total
		Par	Non-par	Par	Non-par	Par	Non-par	
Hypertension experience rate	Urban	64 (4.5%)	704 (49.7%)	339 (23.9%)	429 (30.3%)	640 (45.2%)	128 (9.0%)	768 (54.2%)
	Rural	610 (15.0%)	1496 (36.8%)	867 (21.3%)	1239 (30.5%)	1502 (37.0%)	604 (14.9%)	2106 (51.8%)
Hypertension inexperience rate	Urban	73 (5.2%)	576 (40.6%)	315 (22.2%)	334 (23.6%)	561 (39.6%)	88 (6.2%)	649 (45.8%)
	Rural	743 (18.3%)	1215 (29.9%)	942 (23.2%)	1016 (25.0%)	1405 (34.6%)	553 (13.6%)	1958 (48.2%)
χ^2^		3.422	36.859 ***	2.735	19.799 ***	2.629	0.095	
Diabetes experience rate	Urban	28 (2.0%)	334 (23.6%)	158 (1.2%)	204 (14.4%)	310 (21.9%)	52 (3.7%)	362 (25.5%)
	Rural	189 (4.7%)	476 (11.7%)	281 (6.9%)	384 (9.4%)	484 (11.9%)	181 (4.5%)	665 (16.4%)
Diabetes inexperience rate	Urban	109 (7.7%)	946 (66.8%)	496 (35.0%)	559 (39.4%)	891 (62.9%)	164 (11.6%)	1055 (74.5%)
	Rural	1164 (28.6%)	2235 (55.0%)	1528 (37.6%)	1871 (46.0%)	2423 (59.6%)	976 (24.0%)	3399 (83.6%)
χ^2^		2.081	8.495 **	1.230	1.640	0.291	0.611	
Heart disease experience rate	Urban	35 (2.5%)	509 (35.9%)	226 (15.9%)	318 (22.4%)	453 (32.0%)	91 (6.4%)	544 (38.4%)
	Rural	296 (7.3%)	823 (20.3%)	477 (11.7%)	642 (15.8%)	790 (19.4%)	329 (8.1%)	873 (61.6%)
Heart disease inexperience rate	Urban	102 (7.2%)	771 (54.4%)	428 (30.2%)	445 (31.4%)	748 (52.8%)	125 (8.8%)	1119 (27.5%)
	Rural	1057 (26.0%)	1888 (46.5%)	1332 (32.8%)	1613 (39.7%)	2117 (52.1%)	828 (20.4%)	2945 (72.5%)
χ^2^		10.577 **	32.532 ***	7.550 **	2.223	1.506	0.658	
Stroke experience rate	Urban	14 (1.0%)	167 (11.8%)	65 (4.6%)	116 (8.2%)	152 (10.7%)	29 (2.0%)	181 (12.8%)
	Rural	120 (3.0%)	354 (8.7%)	175 (4.3%)	299 (7.4%)	329 (8.1%)	145 (3.6%)	1236 (87.2%)
Stroke inexperience rate	Urban	123 (8.7%)	1113 (78.5%)	589 (41.6%)	647 (45.7%)	1049 (74.0%)	187 (13.2%)	474 (11.7%)
	Rural	1233 (30.3%)	2357 (58.0%)	1634 (40.2%)	1956 (48.1%)	2578 (63.4%)	1012 (24.9%)	3590 (88.3%)
χ^2^		0.888	15.370 ***	8.759 **	12.525 ***	0.097	1.186	

Note: Par—Participants; Non-par—Non-participants. ** *p* < 0.01, *** *p* < 0.001. The first *p*-value indicates the statistical significance of the difference in disease experience rates between PA participants and non-participants in urban areas, and the second *p*-value indicates the statistical significance of the difference in disease experience rates between PA participants and non-participants in rural areas. This explanation applies to the three intensities of PA.

**Table 2:** Two-way ANOVA for differences in depression symptoms by PA and residential area.

Variables		N	Urban	Rural	F Value
Vigorous PA	Participant	1490	7.270 ± 6.056	10.370 ± 6.505	0.543 74.183 1.074
	Non-participant	3991	7.830 ± 6.080	10.280 ± 6.683	
Moderate PA	Participant	2463	7.170 ± 5.755	10.370 ± 6.718	0.698 17.944 9.232 **
	Non-participant	3018	8.290 ± 6.300	10.270 ± 6.548	
Low PA	Participant	4108	7.410 ± 5.911	10.140 ± 6.586	2.738 4.192 11.503 **
	Non-participant	1373	9.790 ± 6.596	10.730 ± 6.701	

Note: ** *p* < 0.001. In the F-value column, the first row represents the F-value (*p*) for physical activity (PA), the second row represents the F-value (*p*) for residential area, and the third row represents the F-value (*p*) for the interaction between PA and residential area.

**Table 3:** Correlation analysis on urban residence, PA, chronic diseases, and depression symptoms.

Variables	Urban	Vigorous PA	Moderate PA	Low PA	Hypertension	Diabetes	Heart Disease	Stroke	Depression Symptoms
Urban	1								
Vigorous PA	−0.207 **	1							
Moderate PA	0.037 **	0.208 **	1						
Low PA	0.156 **	0.025	0.175 **	1					
Hypertension	0.021	−0.095 **	−0.069 **	−0.011	1				
Diabetes	0.103 **	−0.071 **	−0.025	0.024	0.205 **	1			
Heart Disease	0.103 **	−0.112 **	−0.033 *	−0.005	0.207 **	0.170 **	1		
Stroke	0.015	−0.059 **	−0.062 **	−0.011	0.164 **	0.094 **	0.131 **	1	
Depression symptoms	−0.169 **	0.030 *	−0.027 *	−0.089 **	0.097 **	0.076 **	0.147 **	0.107 **	1

Note * *p* < 0.05 ** *p* < 0.01.

## Data Availability

The data can be accessed through the China Health and Retirement Longitudinal Study website (https://charls.pku.edu.cn/) at Peking University.
